# Elemental Homeostasis Deciphers the Multidimensional Stoichiometric Niche of Fish Communities in the Beibu Gulf

**DOI:** 10.1002/ece3.72611

**Published:** 2025-12-04

**Authors:** Caiguang Wang, Liangliang Huang, Shuwen Zhao, Yiheng Yang, Pengkang She, Jie Chen, Huinan Wu, Xuewen Yang, Shijie Song

**Affiliations:** ^1^ College of Environmental Science and Engineering Guilin University of Technology Guilin China; ^2^ Guangxi Key Laboratory of Environmental Pollution Control Theory and Technology Guilin University of Technology Guilin China; ^3^ University Engineering Research Center of Watershed Protection and Green Development, Guangxi Guilin University of Technology Guilin China

**Keywords:** Beibu Gulf, ecological stoichiometry, fishes, homeostatic, multidimensional stoichiometric niche

## Abstract

The elemental composition of organisms serves as a relatively standardized and easily quantifiable metric, facilitating quantitative analyses that elucidate species coexistence within ecological communities. The recently proposed multidimensional stoichiometric niche (MSN) framework employs the chemical composition of organisms to delineate the ecological niches of various species. This framework presents a perspective for understanding the functional roles and material cycling mechanisms of fish within aquatic ecosystems. To explore the MSN of fish in the Beibu Gulf and its relationship with elemental homeostasis, the content characteristics of carbon (C), nitrogen (N), phosphorus (P), calcium (Ca), potassium (K), iron (Fe), and zinc (Zn) in 46 fish species from 10 families in the Beibu Gulf were analyzed during April and August 2022. The results revealed significant interspecific variations in the elemental composition among fish from different families. These variations resulted in the existence of mutually differentiated and overlapping MSNs between taxonomic groups of different fish families. The differences in elemental content and ecological niches among various fish species are primarily influenced by morphological characteristics and ecological habits. Further analysis uncovered a negative correlation between the MSN of fish and elemental homeostasis in the Beibu Gulf, with species exhibiting higher homeostasis having smaller ecological niches. This reflects the trade‐offs organisms make in response to environmental variability: species with higher homeostasis can reduce the risk of mutation by using fewer resources, while species with lower homeostasis enhance their adaptability through a wider niche. This suggests that organisms can respond to elemental limitations by adjusting their niche width. This study expands the application of ecological stoichiometry theory in fish ecology and has important implications for fishery resource management and ecosystem conservation in the Beibu Gulf.

## Graphical abstract

Significant interspecific variations were observed in the elemental composition among fish from different families in the Beibu dGulf, differentiated or overlapping multidimensional stoichiometric niches exist among taxa of different fish families. The differences in fish elements and ecological niches are mainly affected by their morphological characteristics and ecological habits. The multidimensional stoichiometric niche of fishes in the Beibu Gulf was negatively correlated with elemental homeostasis, and species with higher homeostasis had smaller niches.

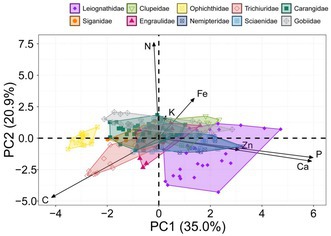

## 1 Introduction

The concept of the ecological niche is the foundation of ecology, offering a descriptive framework for the roles and functions of species within ecosystems, alongside their interactions with both the environment and other species (Ascanio et al. 2024; Sopniewski et al. 2024). Niche theory occupies a central position in explaining how abiotic and biotic factors influence species distribution and abundance, as well as their utilization, transformation, and competition for environmental resources (Kraft et al. 2008; Takola and Schielzeth 2022). Elements are the fundamental substances that constitute living organisms. The elemental content and its proportion (elementome) within an organism are species‐specific, while also reflecting the species' adaptation to its living environment (Sardans et al. 2015; Vallicrosa et al. 2022). With the advances of ecological stoichiometry, the application of the elementome across various research scales within ecology has significantly expanded (Peñuelas et al. 2019; Allgeier et al. 2020). Peñuelas et al. (2008, 2019) and González et al. (2017) proposed the biogeochemical niche hypothesis (BNH), also known as the multidimensional stoichiometric niche (MSN) hypothesis based on the elemental composition of organisms. This hypothesis delineates the ecological niche of a species based on the multiple elements that make up the organisms, facilitating the analysis of species position and volumes of species within the n‐dimensional space composed of multiple element contents, as well as their distinctions from other species. Similar to other traits, elemental composition and its variations can be depicted in a multivariate space, facilitating the visual analysis of an organism's ecological niche and trait distribution. This analysis can be executed at various scales, ranging from individual organisms within a species to entire communities. Such a multiscale approach enables ecologists to gauge the degree to which different levels of biological organization contribute to the overall trophic structure and resource diversity (Peñuelas et al. 2019).

The MSN hypothesis posits that through long‐term evolutionary processes, species develop distinct morphological, physiological, and functional traits, which subsequently shape the demand and distribution of chemical elements within their tissues and organs (González et al. 2017; Peñuelas et al. 2019). However, despite the multi‐elemental nature of organisms, current research in ecological stoichiometry remains largely centered on carbon (C), nitrogen (N), and phosphorus (P). This has resulted in limited exploration into the quantification of high‐dimensional stoichiometric niches, particularly in animal ecosystems. By analyzing soil animal taxa, Zhang et al. (2022) showed that MSNs defined by 10 elements resulted in a lower niche overlap among nine taxonomic groups than those based on C, N and P. Furthermore, the 10‐element MSNs provided a more precise quantification of niche location, size, and overlap across different taxa. Their findings highlight the need to include more elements, beyond the more common C, N, and P, when characterizing the stoichiometric niches of animal taxa.

The MSN hypothesis posits that species vary in their elemental composition and stoichiometric niches, and such differences increase with phylogenetic distance (Peñuelas et al. 2008, 2019; González et al. 2017). In comparison to non‐coexisting species, stably coexisting species tend to differentiate their elementomes to mitigate competitive pressure for elements. In different environments, both stable and fluctuating, there is a trade‐off between homeostasis (which increases competitive ability) and plasticity (which increases success), positioning them along a continuum of strategies (Peñuelas et al. 2019). Consequently, MSN is a comprehensive indicator of taxonomic differences, habitat overlap, and the balance between homeostasis and plasticity. Among these factors, a species' intrinsic requirement for elements shapes its fundamental biogeochemical niche. The n‐dimensional hypervolume of the fundamental niche can be delineated by measuring the elemental composition of multiple individuals within the species. A species' realized niche fluctuates within the n‐dimensional hypervolume of its fundamental niche according to environmental conditions (including abiotic conditions and competition intensity) and homeostatic regulation degree (Yu et al. 2015; Zhang et al. 2025).

González et al. (2017) proposed the creation of a cross‐species chemometric database to improve global comparability of ecological niche parameters. Since then, the MSN hypothesis has been extensively validated and applied across various ecosystems. For example, research on species coexistence in natural forest communities (de la Riva et al. 2017; Sardans et al. 2021), artificial shrub and grassland communities (Urbina et al. 2017), and coastal wetland herbaceous communities (Hu et al. 2018) has demonstrated that species‐specific plant biogeochemical niches differentiate within communities and can adjust to environmental changes and interspecific competition. Furthermore, Sobczyk et al. (2020) found different spider species, as well as male and female individuals within the same species, have unique elemental compositions, occupying different biogeochemical niches that affect the structure and function of spider populations and communities. The C:N:P ecological stoichiometry of freshwater filter‐feeding shellfishes shows distinct niche differences due to their evolutionary history, resulting in niche complementarity and resource differentiation among species (Atkinson et al. 2020). Huang et al. (2023) compared the intraspecific and interspecific differences in the multidimensional stoichiometric niche and carbon and nitrogen stable isotope nutritional niche of three fish species and found that compared with the stable isotope nutritional niche, the multidimensional stoichiometric niche can better reflect the intraspecific differences. González et al. (2017) calculated the MSNs occupied by terrestrial and freshwater food webs, trophic groups, individual species, and individuals within species, and found that the MSNs of vertebrates, invertebrates, and primary producers did not overlap. MSN can be used as a comprehensive approach to assess and compare stoichiometric variations within species, among populations, and across different communities. Stoichiometric data have broad potential for elucidating trophic structure, especially when integrated with other trophic‐related functional traits or community‐level attributes (Leal et al. 2017). This could provide a powerful and unifying framework for assessing niche partitioning and food web structure across diverse ecosystem types and a wide range of biogeographic scales. Furthermore, given the close relationship between nutrient dynamics and the stoichiometry of organisms, the analysis of niche overlap in a multidimensional space can reveal the redundancy or complementarity of species in ecosystem functioning (Carmona et al. 2016). Therefore, the MSN concept has the potential to unify physiological, ecological, and ecosystem approaches to understand the role of life in biogeochemistry. Despite this, there is limited research on the MSN of fishes in aquatic ecosystems. Fish play a significant role in these ecosystems, occupying various layers of the water column due to their ability to migrate actively. They also hold various trophic positions and inhabit different ecological niches within the aquatic ecosystems (Hendrixson et al. 2007). Thus, investigating the variations in fish elemental composition and its driving mechanisms is essential for analyzing how aquatic organisms affect the niches and stability of biological communities within these ecosystems.

The Beibu Gulf, situated in the northwestern region of the South China Sea (105°40′–110°10′ E, 17°00′–21°45′ N), is a semi‐enclosed bay characterized by complex topography and bottom features, as well as abundant fish resources (Wang et al. 2025). Its distinctive geographical location and rich marine resources make it an essential component of both regional and global marine ecosystems. However, recent years have witnessed significant alterations in the structure of its fish community due to high‐intensity fishing, coastal development, and climate change. This necessitates an urgent examination of species adaptation mechanisms from the perspective of element cycles (Deng et al. 2021; Lao et al. 2021). As key consumers in aquatic ecosystems, fish play a central role in material flow and energy transfer. Most existing studies, however, have focused on resource surveys (Wang et al. 2019; Luo et al. 2025; Su et al. 2025) and the biological traits of dominant species (Su et al. 2023; Xiong et al. 2024; Xu et al. 2024), leaving a gap in the integration of stoichiometric and niche‐based approaches.

This study aimed to validate the MSN hypothesis in fish by analyzing the content of 7 elements—C, N, P, calcium (Ca), potassium (K), iron (Fe), and zinc (Zn)—in 46 fish species from 10 families in the Beibu Gulf. The focus was on investigating the differences in these elements among different fish families. Two hypotheses were proposed: (1) the concentrations of these elements, and subsequently the MSNs, vary among different fish families; (2) MSN is affected by elemental homeostasis, with species maintaining more stable elements likely to have smaller ecological niches. This study provides new empirical support for the MSN hypothesis in fish and serves as a reference for the sustainable development, utilization and scientific management of fishery resources in the Beibu Gulf.

## 2 Material and Methods

### 2.1 Sampling

**FIGURE 1:**
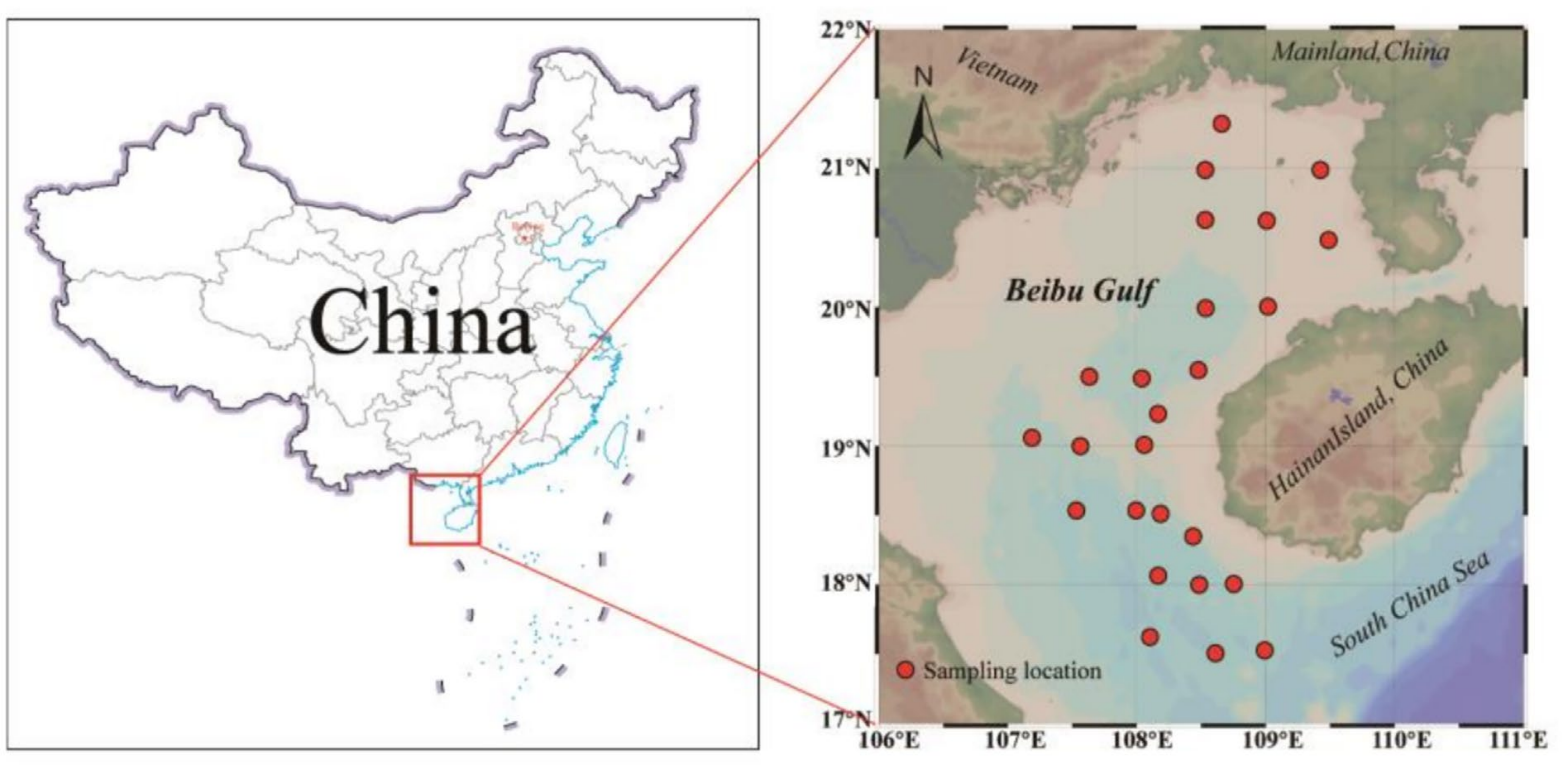
Distribution of sampling sites in the Beibu Gulf. The base map of China was obtained from the National Platform for Common GeoSpatial Information Services (https://www.tianditu.gov.cn/).

A total of 207 fish samples, belonging to 46 species and 10 families, were collected from the Beibu Gulf in April and August of 2022 (Figure 1) using bottom trawl. The main engine power of the ship was 441 kW, the length of the trawl frame was 20 m, the width was 8 m, the height was 3 m, and the mesh sizes were 1–5 cm. All samples were stored at −20°C and shipped to the laboratory for further analysis.

### 2.2 Sample Preparation and Element Determination

Following thawing, the fish samples were rinsed with ultrapure water. The total length (±1 mm), body length (±1 mm) and body weight (±0.1 g) of the samples were measured. To minimize potential bias from gut contents, all individuals were dissected to remove visceral organs following established protocols (Pilati and Vanni 2007; El‐Sabaawi et al. 2014; Allgeier et al. 2021). Subsequently, the samples were freeze‐dried, ground, thoroughly mixed and sieved through a 60‐mesh filter in preparation for chemical analysis. A dry sample weighing between 2.5 and 4.0 mg was analyzed using an elemental analyzer (EA2400II) to determine the C and N content. Then, a 0.2 g dry sample was digested with 7 mL of a mixed acid solution (HNO_3_:H_2_O_2_ = 5:2) by microwave digestion. The digested solution was subsequently heated on a hot plate at 100°C for 30 min to remove residual acid, cooled, and diluted to a final volume of 50 mL with ultrapure water. The concentrations of Ca, P, K, Fe and Zn were determined using inductively coupled plasma emission spectrometry (Optima 7000DV). The recoveries of Ca, P, K, Fe, and Zn ranged from 90% to 110%.

### 2.3 Statistical Analyses

The concentrations of C, N, P, Ca, K, Fe, and Zn in all fish samples were initially subjected to a basic statistical analysis. The Kolmogorov–Smirnov (K‐S) test was used to assess the normality of the data distribution, and a normal histogram was generated to visualize the distribution trend. A one‐way analysis of variance (ANOVA) was conducted to compare the concentrations of these elements among fish from different families. To quantify MSNs and examine niche differentiation among fish families, hypervolumes were calculated using all seven elements via the R package ‘hypervolume’ (Blonder et al. 2018). A principal component analysis (PCA) was initially performed using the R package ‘stats’, and the first three principal component axes were extracted. The distances among hypervolume centroids and the degree of similarity/overlap among the 10 taxa in multivariate space were then determined using the Jaccard similarity index. Using this approach, we can reduce any n elements to three principal components that are generally able to explain most of the variation in the data (i.e., the first three PCA axes) and allow us to calculate the corresponding indices. To test the sensitivity of our results to the number of principal components retained, we performed additional analyses with varying numbers of components. The results indicated that the key patterns of niche size and overlap were robust, even with a reduced number of components. In this study, stoichiometric stability, S (i.e., elemental homeostasis), is defined as the degree of constancy of a variable relative to its mean, expressed as *μ*/*σ*, where *μ* represents the mean value of an element and *σ* denotes its standard deviation, as referenced in Tilman et al. (2006) and Feng et al. (2023). To explore the relationship between the stoichiometric stability of organisms and MSN, Pearson correlation analysis and univariate linear regression were employed to assess the correlation and quantify the proportion of variance explained. All statistical analyses were conducted using SPSS version 23.0, while graphical representations were generated using OriginPro 2021 and R version 4.0.3.

## 3 Results

### 3.1 Element Composition and Differences of Fish From Different Families

In this study, a total of 46 fish species (*n* = 207) belonging to 7 orders, 10 families and 31 genera were collected and analyzed. The mean concentrations of C, N, P, Ca, K, Fe, and Zn were found to be 456,001.061 ± 36,130.552 mg/kg, 114,482.621 ± 13,657.678 mg/kg, 10,576.412 ± 5040.869 mg/kg, 25,320.592 ± 11,340.387 mg/kg, 8398.851 ± 3130.246 mg/kg, 57.405 ± 32.642 mg/kg and 47.566 ± 25.308 mg/kg, respectively. The stoichiometric stability values (S) for these elements were calculated as 19.718, 13.158, 6.0773, 4.397, 3.691, 2.326, and 4.281, respectively (Table 1).

**TABLE 1:** Contents and stoichiometric stability (S) of C, N, P, Ca, K, Fe, and Zn in fishes from different families in the Beibu Gulf.

Items	Statistics	Gobiidae	Nemipteridae	Sciaenidae	Engraulidae	Clupeidae	Leiognathidae	Carangidae	Siganidae	Ophichthidae	Trichiuridae
C (mg/kg)	Mean ± SD	430,979.310 ± 18,246.483	433,900.611 ± 17,403.607	440,896.296 ± 23,242.062	448,189.907 ± 26,239.637	451,243.294 ± 24,302.347	454,239.052 ± 48,902.548	457,880.459 ± 21,712.217	471,555.556 ± 21,299.713	492,660.000 ± 19310.630	521,930.769 ± 32,044.900
	S	23.389	24.932	18.970	17.081	18.568	9.2089	21.089	22.139	25.512	16.287
N (mg/kg)	Mean ± SD	127,713.793 ± 5911.171	108,807.249 ± 7590.681	111,651.852 ± 8674.027	114,086.047 ± 15,770.717	123,387.837 ± 7629.299	103,637.480 ± 19,935.921	118,474.844 ± 8898.864	110,155.556 ± 5878.161	112,520.000 ± 7501.970	98,423.077 ± 13,842.817
	S	21.605	14.334	12.872	7.234	16.173	5.199	13.313	18.740	14.999	7.110
P (mg/kg)	Mean ± SD	14,439.227 ± 4412.227	22,019.580 ± 3855.853	18,277.043 ± 3047.068	14,382.200 ± 2629.159	17,725.948 ± 3369.133	23,290.739 ± 4085.497	14,929.548 ± 2048.814	14,608.218 ± 2676.201	8593.963 ± 810.240	12,506.904 ± 2082.398
	S	3.273	5.711	5.998	5.470	5.261	5.701	7.287	5.459	10.607	6.006
Ca (mg/kg)	Mean ± SD	23,318.528 ± 9211.479	36,538.399 ± 8479.373	34,087.406 ± 9571.443	21,476.741 ± 3416.957	25,444.979 ± 5475.851	38,294.872 ± 9364.293	19,065.381 ± 5061.824	19,221.832 ± 4015.657	7604.124 ± 1584.986	17,697.740 ± 3407.277
	S	2.531	4.309	3.561	6.285	4.647	4.089	3.767	4.787	4.798	5.194
K (mg/kg)	Mean ± SD	8307.242 ± 2962.416	8162.457 ± 2236.433	10,120.439 ± 2259.276	7979.099 ± 3811.695	9988.318 ± 5255.166	6839.966 ± 2036.765	8334.258 ± 2043.583	6500.657 ± 2630.768	9023.389 ± 960.219	5306.976 ± 1981.790
	S	2.804	3.650	4.480	2.093	1.901	3.358	4.078	2.471	9.397	2.678
Fe (mg/kg)	Mean ± SD	58.971 ± 30.909	49.837 ± 16.218	51.884 ± 19.942	56.461 ± 35.463	74.543 ± 50.369	47.783 ± 34.840	73.527 ± 30.917	60.666 ± 12.405	31.851 ± 12.368	48.811 ± 35.137
	S	1.908	3.073	2.602	1.592	1.480	1.371	2.378	4.890	2.575	1.389
Zn (mg/kg)	Mean ± SD	35.675 ± 8.154	30.273 ± 6.542	37.688 ± 9.669	59.178 ± 17.199	79.724 ± 27.887	84.059 ± 29.467	42.990 ± 10.437	37.473 ± 7.833	34.020 ± 4.453	26.055 ± 6.190
	S	4.375	4.627	3.898	3.441	2.859	2.853	4.119	4.784	7.640	4.209

**FIGURE 2:**
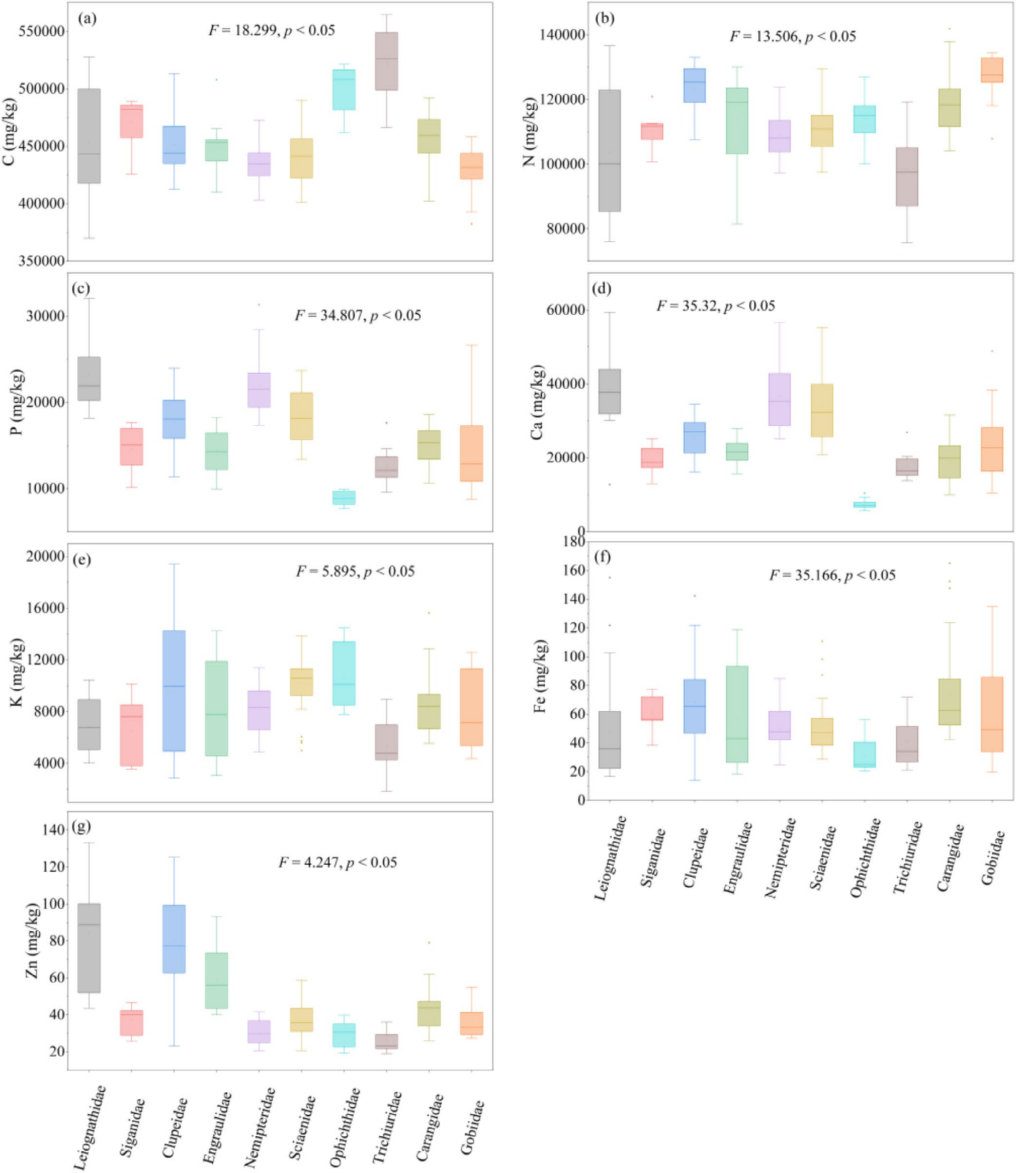
Box plot of (a) C, (b) N, (c) P, (d) Ca, (e) K, (f) Fe and (g) Zn content in different families of fish from the Beibu Gulf (different lowercase letters indicate significant differences (*p* < 0.05)).

The variance analysis results revealed significant differences in the contents of C, N, P, Ca, K, Fe, and Zn among fish from different families (Figure 2). Specifically, Ophichthidae and Trichiuridae exhibited the highest C levels, whereas Gobiidae and Nemipteridae presented the lowest when compared to several other families. Moreover, the N content was found to be the highest in the Clupeidae and Gobiidae. The highest concentrations of P and Ca were observed in Leiognathidae and Nemipteridae, with Ophichthidae having the lowest. The K content was predominantly present in Sciaenidae and Ophichthidae, while its concentration was lowest in Trichiuridae. The content of Fe in Ophichthidae was significantly lower than that in Siganidae, Nemipteridae, Sciaenidae, Carangidae, and Gobiidae. Conversely, the Fe content in Carangidae was significantly higher than that in Nemipteridae, Sciaenidae, Ophichthidae and Trichiuridae. The content of Zn in Leiognathidae, Clupeidae and Engraulidae was significantly higher than that in other families.

### 3.2 Multidimensional Stoichiometric Niches of Fishes From Different Families

**FIGURE 3:**
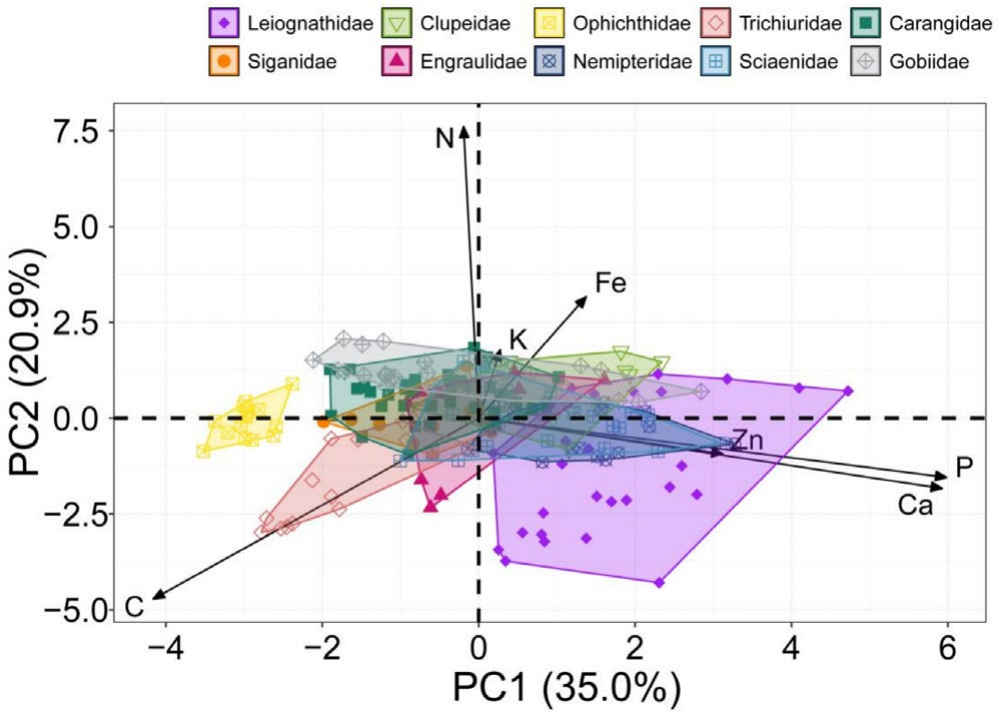
Multi‐element principal component analysis (PCA) of fishes from different families from the Beibu Gulf.

PCA was applied to examine the characteristic relationships among multidimensional stoichiometric niches (MSNs) across different fish families. The first two principal components (PC1 and PC2) accounted for 50.9% of the total variation in the contents of seven elements in fish. Notably, PC1 exhibited the most significant loading on C, P, and Ca contents, while PC2 had its highest loading on N content (Table 2). When plotted along these PCA axes, fishes from different families were distinctly separated, with the majority exhibiting niche overlaps (Figure 3). Of these, Leiognathidae possessed the most extensive niche hypervolume, followed by Engraulidae, Clupeidae, Trichiuridae, Sciaenidae, Gobiidae, Siganidae, Nemipteridae, and Carangidae, while Ophichthidae had the smallest niche hypervolume (Table 3). The probability of overlap between Sciaenidae and Nemipteridae was the largest. Conversely, the overlap probability between Trichiuridae and other families was relatively low, with the exception of Siganidae. Notably, there was an absence of niche overlap between Ophichthidae and all other families.

**TABLE 2:** Distribution of multi‐element principal component loadings of fishes from different families from the Beibu Gulf.

Element	PC1	PC2	PC3
C	−0.416	−0.472	0.058
N	−0.019	0.761	0.028
P	0.598	−0.154	0.060
Ca	0.593	−0.183	−0.025
K	0.028	0.178	0.889
Fe	0.137	0.317	−0.431
Zn	0.312	−0.093	0.121
Total variation explained	35.0%	20.9%	14.7%

**TABLE 3:** Hypervolume niche size (the last column), pairwise niche distance (the upper triangular panel) and niche overlap (the lower triangular panel) of different families of fishes from the Beibu Gulf.

Species	Leiognathidae	Siganidae	Clupeidae	Engraulidae	Nemipteridae	Sciaenidae	Ophichthidae	Trichiuridae	Carangidae	Gobiidae	hypervolume
Leiognathidae		2.982	2.187	2.279	0.992	1.610	5.101	3.722	2.967	3.040	146.245
Siganidae	0.092		2.139	1.105	2.274	1.984	2.818	1.732	0.945	1.583	40.437
Clupeidae	0.171	0.280		1.123	1.226	0.695	3.813	3.565	1.452	1.167	67.087
Engraulidae	0.232	0.240	0.385		1.424	0.936	3.064	2.446	0.714	1.151	122.856
Nemipteridae	0.224	0.186	0.250	0.178		0.697	4.406	3.378	2.070	2.060	35.746
Sciaenidae	0.217	0.239	0.345	0.291	0.464		3.819	3.184	1.554	1.584	52.744
Ophichthidae	0.000	0.003	0.000	0.000	0.000	0.000		2.707	2.691	3.313	9.062
Trichiuridae	0.046	0.316	0.115	0.171	0.073	0.058	0.024		2.478	3.248	52.972
Carangidae	0.070	0.391	0.254	0.174	0.192	0.253	0.017	0.129		0.812	25.678
Gobiidae	0.119	0.159	0.311	0.251	0.136	0.239	0.023	0.039	0.268		51.945

### 3.3 Correlation Between Multidimensional Stoichiometric Niche and Stoichiometric Stability in Fishes of Different Families

**FIGURE 4:**
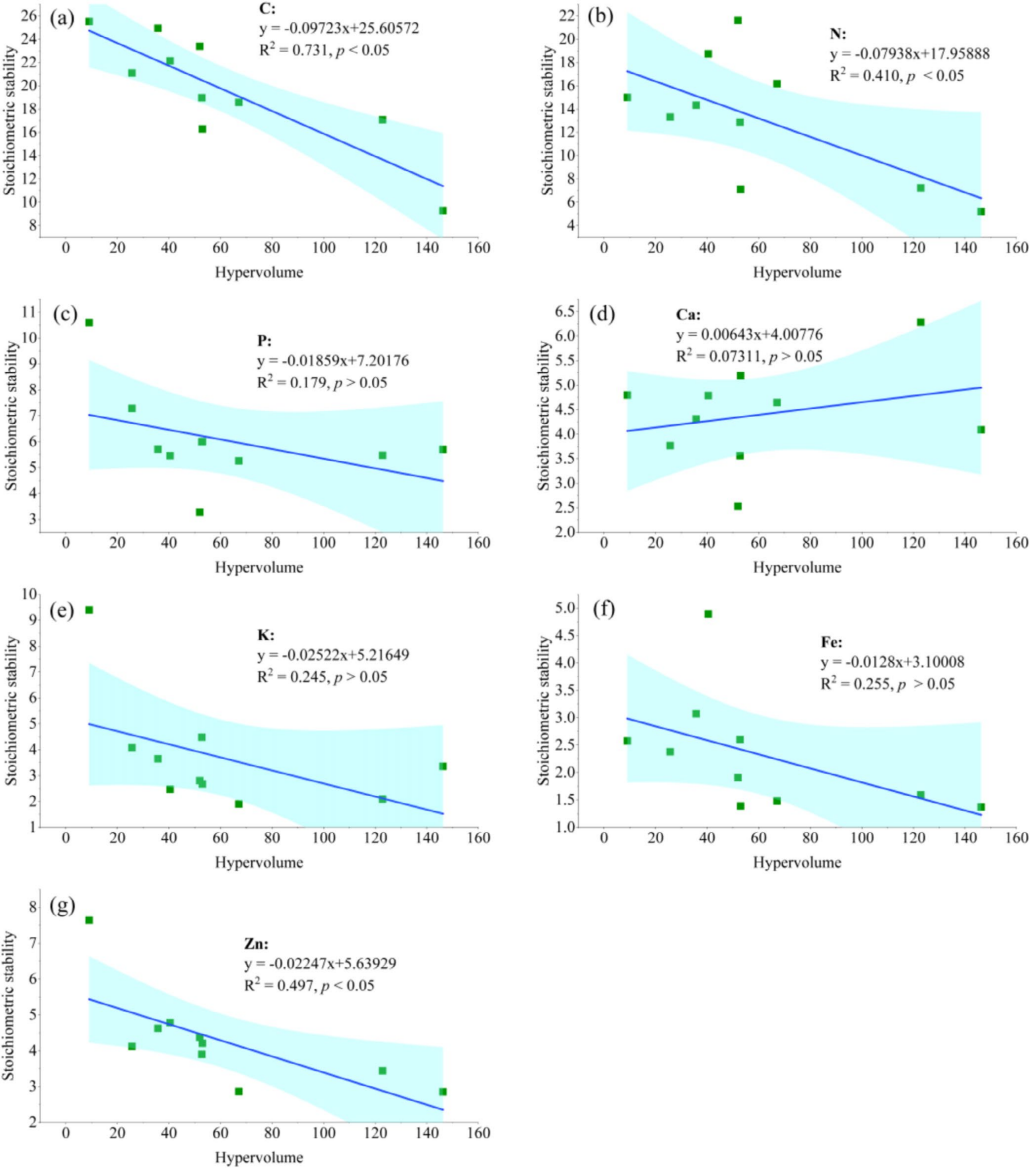
Correlation analysis between multidimensional stoichiometric niche (MSN) hypervolume and stoichiometric stability of fish from the Beibu Gulf.

The correlation analysis revealed a significant negative relationship between the concentrations of C, N, and Zn in fish from the Beibu Gulf and their niche size (*R* ^2^ = 0.731, *R* ^2^ = 0.410, *R* ^2^ = 0.497 *p* < 0.05). With the exception of Ca, the stoichiometric stability (S) of other elements showed a decreasing trend as the increase in ecological niche (Figure 4).

## 4 Discussion

### 4.1 Element Composition and Differences of Fish From Different Families

Biological communities exhibit a consistent elemental composition, and these characteristics can be utilized to evaluate the similarities or differences between different taxa (Leal et al. 2017). Our study extended the scope of ecological stoichiometry beyond the traditional focus on C, N, and P elements, elucidating distribution patterns of elements such as C, N, P, Ca, K, Fe, and Zn in marine organisms. C is the primary constituent element of all organic substances, while N and P are fundamental structural elements for all organisms. Ca and K are vital for all animals and plants, essential for the functioning of ion channels that regulate ion passage across cell membranes. Notably, Ca is the primary inorganic substance in bones (Horrigan and Aldrich 2002; Zhang, Chang, et al. 2023). Fe and Zn are important essential trace elements with diverse biochemical functions (Sharma et al. 2021). At low concentrations, metals like Fe and Zn are crucial for enzyme activity and various biological processes in fish (Yilmaz 2003). This study determined a hierarchical concentration of elements in fish as C > N > Ca > P > K > Fe > Zn, with significant variability observed across different fish families. The elemental composition in fish reflects the elemental composition of their habitats and correlates with their biological utilization and nutritional requirements (Andrieux et al. 2020; Atkinson et al. 2020). Species‐specific biochemical structures and physiological metabolic processes lead to distinct intrinsic elemental needs, resulting in interspecies variations in body elemental composition between species (Andrieux et al. 2020). The most accurate predictor of ecological stoichiometry is the taxonomic classification at the family level. It's worth noting that the ecological stoichiometric characteristics of fish exhibit considerable variability at the family level but less so within families (Hendrixson et al. 2007; Allgeier et al. 2020, 2021; Wang et al. 2023).

The variations in the elemental composition among organisms are largely determined by the enrichment of specific components with distinct elemental contents. For example, cellulose and lignin that are C‐rich in plants, chitin that is rich in C and N in arthropods, and muscle and bone that are rich in P in vertebrates. These components exhibit distinct distributions and proportions across different organisms, and their structural and morphological features are also quite variable (Elser et al. 2003; Leal et al. 2017). For instance, C‐rich lipids and N‐rich muscles have a dilution effect on the concentrations of Ca and P due to the increase in the proportion of lipids and muscles, respectively, which affects the overall Ca and P ratios in the organisms (Elser et al. 1996; Boros et al. 2015). Fishes exhibit more variation in body shape than other vertebrates, and their stoichiometric characteristics are likely to be more variable with body morphology (Vrede et al. 2011). This relationship arises from differences in the distribution of muscle, bone, and scales across fish with distinct body shapes, each tissue type possessing unique structural properties linked to specific biological functions. In this study, fishes from different families had significantly different body shapes, and the contents of C, N, P, and other elements also varied greatly among them. Moreover, the Ca and P contents in eel‐type fishes were found to be significantly lower than those in other fishes. This was mainly related to the smaller surface area‐to‐volume and head‐to‐total length ratios of the eel‐type fishes, their high body muscle content, and the absence of scales on their bodies. The K, Fe, and Zn contents also differed significantly among fish families, being affected by species and anatomical part among other factors. The K content was found to be higher in whole fish samples than that in muscle samples, as bones and other tissues contain K (Leng et al. 2024). Zn is distributed unevenly in fish, with higher concentrations found in viscera and muscle, and lower concentrations in fat and gills (Yaqoob et al. 2020). The K, Fe, and Zn contents differ according to fish size; for instance, the K content increases with body weight in *Saurida tumbil* and *Pennahia macrocephalus* , while Fe and Zn contents decline (Huang et al. 2023). Furthermore, fish from different families show differences in elemental composition depending on their diets, and differences may also exist between seasons. Fish living conditions, such as water temperature, climate, water quality, and food availability, can affect their growth, development, and morphology, leading to variations in the content of elements like C, N, P, and Ca. Similarly, seasonal variations in food availability and increased fat content during spawning season can also contribute to variations in these elemental concentrations. Marine fish have a wide variety of diets, which can lead to significant differences in elemental content. Consequently, the variations in elemental compositions among different fish species may be attributed to their diverse dietary habits. Hendrixson et al. (2007) demonstrated that the P content in freshwater fish was related to dietary habits and that carnivorous fish exhibit significantly higher levels of P than omnivorous fish. This study also indicated that the C content in carnivorous fish was much higher than that in herbivorous and omnivorous fish, while the N and P contents in omnivorous fish were significantly higher than those in herbivorous and carnivorous fish. In this study, Leiognathidae and Clupeidae were predominantly classified as omnivorous, while Engraulidae were classified as filter‐feeding species. The Zn content in these families was also significantly higher than that observed in other fish families.

### 4.2 Differences in Fish Niches and Their Influencing Factors

All individuals within an ecological community are composed of the same chemical elements, and these common elemental compositional characteristics can be used to quantify trait similarity or dissimilarity among various taxa (Sterner and Elser 2002; Zhang, Chen, et al. 2023). This research expands the application of ecological stoichiometry to fish niches by highlighting large disparities in body element content and significant niche differentiation among fish in the Beibu Gulf. This study found the existence of mutually differentiated or overlapping MSNs among different fish taxa in the Beibu Gulf. It also uncovered a general trend wherein the niche hypervolume was largest for omnivorous fish, followed in descending order by filter‐feeding fish, herbivorous fish and carnivorous fish. Such niche differentiation facilitates species coexistence by modifying resource use, reducing competition, and enhancing ecosystem stability (Carruthers et al. 2022). A smaller degree of niche overlap suggests less competition and a more stable ecosystem, whereas greater overlap signifies intense resource competition (Peel et al. 2019). The study found no niche overlap between Ophichthidae and other fish families, noting that Ophichthidae have lower concentrations of Ca, P, Fe, and Zn but higher contents of C, N, and K than other families. Ophichthidae are elongated, scaleless fish inhabiting shallow sandy or muddy waters, with primary activity and reproduction occurring at night (Froese and Pauly 2012). Similarly, Trichiuridae, characterized by their ribbon‐like body with smooth skin, inhabit areas near muddy shores and migrate to shallow seas for reproduction, evidencing significant niche differentiation from other fish families (Froese and Pauly 2012). Additionally, Leiognathidae exhibit a notably larger MSN hypervolume, which can be attributed to their specific living habits. They exhibit diverse fish diets and occupy the upper, middle and lower water layers (Froese and Pauly 2012): For example, *Leiognathus brevirostris* (upper and middle layers) and *Photopectoralis bindus* (bottom layer) are omnivorous, whereas *Leiognathus equula* (lower and middle layers), *Nuchequula nuchalis* (lower and middle layers) and *Nuchequula blochii* (bottom layer) are carnivorous. In summary, the variation in element content and ecological niche among different fish species is mainly influenced by their morphological characteristics and ecological habits. These differences have important implications for understanding fish nutritional physiology and ecosystem nutrient cycling.

Research has shown that as stoichiometric stability (S) increases, the dynamic equilibrium of species becomes more stable. It is suggested that each species has an optimal balance of bio‐elemental composition that maximizes fitness. Interestingly, various taxa display distinct elemental contents, with disparities escalating as taxonomic distance and evolutionary time grow (Peñuelas et al. 2019). Furthermore, species exhibit flexibility and adaptability, adjusting their elemental stoichiometry in response to shifts in the composition of neighboring species or environmental conditions (Sardans et al. 2011; Sardans and Peñuelas 2014). Stoichiometric stability refers to an organism's ability to maintain a relatively stable chemical composition by adjusting the ratios of elements, such as C, N, P, Ca, etc., under varying environmental conditions (Feng et al. 2023; Zuo et al. 2024). The study revealed significant differences in stoichiometric stability among species, which correlated closely with the ecological niche, function and stability of those species. For example, Zhang et al. (2022) investigated the stoichiometric niches of nine soil animals and found that the omnivorous group Formicidae exhibited the largest hypervolumetric niche while its elemental composition had a lower homeostasis, a finding that aligns with the results of this study. This study found a negative correlation between the MSN of fish in the Beibu Gulf and elemental homeostasis, indicating that species with greater stability had smaller ecological niches. In other words, the larger the S, the smaller the MSN hypervolume. This also indicates that the MSN serves as a reflection of species homeostasis, an attribute that allows them to retain relatively stable elemental compositions despite fluctuating environmental conditions (Yu et al. 2015). This reflects the trade‐offs organisms make in response to environmental change: species with higher homeostasis can reduce the risk of mutation by using fewer resources, while species with lower homeostasis enhance their adaptability through a wider niche. This suggests that organisms respond to elemental limitations by adjusting their niche width. Moreover, intraspecific variation could potentially influence MSN. The greater the intraspecific variation, the less homeostasis the species can uphold. A study by Huang et al. (2023) corroborated this point by demonstrating that increased intraspecific differences correspond to a larger MSN in the species, reaffirming the negative correlation between MSN and homeostasis. In addition, MSN is related to individual development. Relatively large individuals have smaller MSNs. This study found Leiognathidae, which exhibited smaller body sizes, to have broader ecological niches. Secondly, the correlation results between Ca homeostasis and MSN in this study were inverse to those of other elements, indicating that fish are not limited by Ca during growth. We speculate that the possible reason is that Ca is the main mineral component of fish bones and scales. The demand for Ca is relatively high during the individual development of fish. The ecological environment of the Beibu Gulf has always had sufficient Ca to support the growth and development of fish (Huang et al. 2018), so they do not need to adjust the Ca content to adapt to environmental changes.

## 5 Conclusion

This study's findings reveal significant interspecific variations in the elemental composition among different fish families in the Beibu Gulf. Distinct or overlapping multidimensional stoichiometric niches were identified across these families. Leiognathidae exhibited the largest niche hypervolume, followed in descending order by Engraulidae, Clupeidae, Trichiuridae, Sciaenidae, Gobiidae, Siganidae, Nemipteridae, and Carangidae. Conversely, Ophichthidae displayed the smallest niche hypervolume. Overall, the niche hypervolumes displayed a trend of omnivorous fishes > filter‐feeding fishes > herbivorous fishes > carnivorous fishes. The differences in fish elements and ecological niches are mainly affected by the morphological characteristics and ecological habits. Additionally, the MSN of fish was found to be negatively correlated with elemental homeostasis, indicating that species with greater stability possess smaller niches. Specifically, as the S increases, the MSN hypervolume decreases. This study expands the application of ecological stoichiometry theory in fish ecology. Fish serve as excellent vertebrate models for such research, but further studies examining other animals, as well as intraspecific differences in the MSNs of species at different developmental stages, are still necessary.

## Author Contributions

**Caiguang Wang:** methodology (equal), visualization (equal), writing – original draft (equal), writing – review and editing (equal). **Liangliang Huang:** conceptualization (equal), resources (equal), supervision (equal), writing – review and editing (equal). **Shuwen Zhao:** data curation (equal), investigation (equal), writing – original draft (equal). **Yiheng Yang:** investigation (equal), writing – original draft (equal). **Pengkang She:** investigation (equal), writing – original draft (equal). **Jie Chen:** investigation (equal), writing – original draft (equal). **Huinan Wu:** investigation (equal), writing – original draft (equal). **Xuewen Yang:** investigation (equal), writing – original draft (equal). **Shijie Song:** conceptualization (equal), formal analysis (equal), project administration (equal), writing – review and editing (equal).

## Funding

This work was supported by Guangxi Science and Technology Program, Guike AD25069074; Key Research and Development Program of Guangxi, Guike AB22035050; Innovation Project of Guangxi Graduate Education, YCBZ2025172.

## Conflicts of Interest

The authors declare no conflicts of interest.

## Supporting information


**Data S1:** ece372611‐sup‐0001‐supinfo.docx.

## Data Availability

All the required data is uploaded as Supporting Information.
