# Supplementary material for: Elemental Homeostasis Deciphers the Multidimensional Stoichiometric Niche of Fish Communities in the Beibu Gulf
Source: Ecol Evol. 2025 Dec 4;15(12):e72611. doi: 10.1002/ece3.72611 (PMC12676182; doi:10.1002/ece3.72611)
Supplement: Supplementary file 1 — Data S1: ece372611‐sup‐0001‐supinfo.docx. [file ECE3-15-e72611-s001.docx]

Supplemental files

**Raw data:**

| taxon | body weight (g) | C (mg/kg) | N (mg/kg) | P (mg/kg) | Ca (mg/kg) | K (mg/kg) | Fe (mg/kg) | Zn (mg/kg) |
| --- | --- | --- | --- | --- | --- | --- | --- | --- |
| Leiognathidae | 15.31 | 516200 | 95900 | 23794.06653 | 43952.95432 | 7667.576961 | 74.23038729 | 94.46375372 |
| Leiognathidae | 20.99 | 501400 | 88600 | 21418.86931 | 38287.44493 | 7558.737151 | 155.1639745 | 93.979442 |
| Leiognathidae | 16.02 | 481900 | 98200 | 24409.39514 | 43918.56718 | 9366.633614 | 36.44025781 | 88.99355478 |
| Leiognathidae | 17.11 | 446900 | 106400 | 26529.89665 | 46602.48524 | 10435.5315 | 39.49311024 | 83.90748031 |
| Leiognathidae | 133.75 | 417600 | 108700 | 20743.19307 | 34791.4604 | 4356.435644 | 31.93069307 | 47.8960396 |
| Leiognathidae | 108.76 | 435300 | 102000 | 25254.83631 | 12780.87798 | 4861.731151 | 47.49503968 | 49.6031746 |
| Leiognathidae | 138.31 | 440200 | 103800 | 21718.90364 | 37059.36578 | 4792.895772 | 24.21337266 | 43.633235 |
| Leiognathidae | 97.67 | 423800 | 94800 | 28239.06017 | 50511.56141 | 5215.688712 | 16.65837892 | 48.60765788 |
| Leiognathidae | 116.32 | 425700 | 107600 | 22665.64792 | 38657.09046 | 5301.344743 | 20.17114914 | 46.57701711 |
| Leiognathidae | 8.58 | 377422.1128 | 125216.3282 | 24411.39871 | 41382.52862 | 4017.546043 | 72.79741165 | 133.1508213 |
| Leiognathidae | 16.72 | 370082.7888 | 120230.9282 | 32097.51946 | 43743.92023 | 5870.622568 | 79.5233463 | 120.0145914 |
| Leiognathidae | 12.91 | 375945.6418 | 122866.4582 | 33422.09073 | 59399.65483 | 6947.115385 | 121.7948718 | 130.6706114 |
| Leiognathidae | 12.78 | 404405.6438 | 130260.8272 | 21319.85294 | 35355.39216 | 4762.867647 | 102.6960784 | 104.1666667 |
| Leiognathidae | 33.91 | 497300 | 82400 | 18878.98804 | 30059.82054 | 8927.592223 | 22.43270189 | 50.22432702 |
| Leiognathidae | 19.95 | 527700 | 81400 | 19117.96799 | 30407.37148 | 9333.777886 | 17.70126091 | 62.68186227 |
| Leiognathidae | 19.43 | 499600 | 82600 | 20222.15496 | 32687.65133 | 9653.1477 | 17.79661017 | 58.11138015 |
| Leiognathidae | 25.23 | 505900 | 92900 | 21018.90756 | 33063.51953 | 10471.45329 | 19.15472071 | 51.90311419 |
| Leiognathidae | 16.61 | 483700 | 76000 | 19483.82782 | 30970.33074 | 9537.329767 | 21.15758755 | 65.6614786 |
| Leiognathidae | 12.2 | 406295.6928 | 132166.1372 | 23049.70617 | 38479.43193 | 7291.25857 | 40.52399608 | 88.14887365 |
| Leiognathidae | 11.75 | 414287.5348 | 134145.8282 | 21399.40974 | 36257.99311 | 6542.67093 | 33.32513527 | 94.44171176 |
| Leiognathidae | 15.81 | 430351.2988 | 134286.5382 | 20257.47916 | 31896.76312 | 7062.89848 | 28.44531633 | 90.73075037 |
| Leiognathidae | 11.85 | 433124.6408 | 136601.4362 | 18169.77429 | 31446.27085 | 7158.366045 | 39.86751717 | 130.642787 |
| Leiognathidae | 16.04 | 519500 | 78600 | 29497.87924 | 53786.8014 | 5568.862275 | 35.80339321 | 100.0499002 |
| Leiognathidae | 17.34 | 488000 | 85500 | 22156.14498 | 38982.83725 | 4950.192031 | 49.32789246 | 95.41526644 |
| Leiognathidae | 13.78 | 467500 | 93900 | 27256.70732 | 49720.12195 | 5062.195122 | 61.82926829 | 129.1463415 |
| Leiognathidae | 12.46 | 520100 | 79500 | 19025.53763 | 31464.44282 | 5124.633431 | 32.38025415 | 82.72238514 |
| Siganidae | 86.18 | 488200 | 100700 | 15098.58631 | 18758.06052 | 8518.725198 | 65.35218254 | 27.77777778 |
| Siganidae | 59 | 425800 | 120900 | 15705.42731 | 17395.01473 | 10141.8222 | 56.11493124 | 28.85559921 |
| Siganidae | 53.54 | 482200 | 107700 | 17099.72946 | 22819.10969 | 8072.429907 | 38.3669454 | 25.70093458 |
| Siganidae | 31.08 | 455300 | 109400 | 16967.04491 | 21054.4423 | 8829.671656 | 77.37807822 | 40.07725736 |
| Siganidae | 38.04 | 457800 | 103400 | 17677.87286 | 25227.99511 | 7627.750611 | 55.86797066 | 42.4205379 |
| Siganidae | 74.58 | 489100 | 111800 | 10123.67278 | 12990.46815 | 3581.684363 | 73.11776062 | 42.3503861 |
| Siganidae | 96.96 | 484600 | 112200 | 11281.97394 | 14733.9527 | 4292.350386 | 56.46718147 | 39.69594595 |
| Siganidae | 77.23 | 475300 | 112600 | 14770.52239 | 22545.39801 | 3623.756219 | 72.01492537 | 46.76616915 |
| Siganidae | 80.75 | 485700 | 112700 | 12749.13022 | 17472.04274 | 3817.718688 | 51.31709742 | 43.61332008 |
| Clupeidae | 77.76 | 475700 | 124200 | 14320.21792 | 16628.3293 | 11130.14528 | 65.73849879 | 63.92251816 |
| Clupeidae | 19.67 | 421200 | 123400 | 14349.52783 | 16830.26839 | 11994.90557 | 78.15606362 | 74.30417495 |
| Clupeidae | 17.8 | 458100 | 124800 | 15551.22361 | 19619.72169 | 11460.53263 | 57.34165067 | 67.65834933 |
| Clupeidae | 20.86 | 413000 | 132000 | 19989.7541 | 28682.49759 | 13534.83607 | 102.0973963 | 99.68659595 |
| Clupeidae | 29.88 | 444200 | 119400 | 17199.97595 | 23232.32323 | 13093.43434 | 48.94179894 | 74.19432419 |
| Clupeidae | 16.02 | 478800.4068 | 129578.3712 | 16289.93224 | 23578.17038 | 4546.829622 | 275.1694095 | 109.7531462 |
| Clupeidae | 20.57 | 471914.7968 | 118967.5902 | 18535.89109 | 28892.32673 | 6968.440594 | 142.3267327 | 118.9356436 |
| Clupeidae | 17.36 | 513220.8188 | 125848.6232 | 18425.18703 | 28485.03741 | 6528.054863 | 242.8927681 | 125.3117207 |
| Clupeidae | 23.81 | 480789.8038 | 111476.9082 | 18421.25 | 28081.25 | 8800.625 | 65.125 | 31.5 |
| Clupeidae | 19.42 | 443740.0578 | 109285.2392 | 11491.20603 | 18486.1809 | 4844.849246 | 67.96482412 | 23.11557789 |
| Clupeidae | 7.64 | 453000 | 127800 | 11363.57963 | 16171.36795 | 2846.355467 | 35.8212681 | 96.3554668 |
| Clupeidae | 8.24 | 440100 | 129600 | 16456.79468 | 24958.76416 | 4541.482029 | 13.78631216 | 88.62629247 |
| Clupeidae | 7.9 | 424800 | 126100 | 18635.41153 | 28766.01774 | 4433.218334 | 17.74273041 | 112.6170527 |
| Clupeidae | 6.97 | 442800 | 133100 | 17716.45289 | 25939.19586 | 5022.816971 | 50.93734583 | 99.16132215 |
| Clupeidae | 5.6 | 441900 | 130200 | 16144.20254 | 23687.01076 | 5026.908023 | 39.62818004 | 93.5665362 |
| Clupeidae | 18.83 | 429200 | 129800 | 23937.46917 | 34428.95905 | 19417.24223 | 90.03453379 | 66.23088308 |
| Clupeidae | 14.81 | 463100 | 107500 | 20548.79548 | 30494.10029 | 15005.53097 | 44.49360865 | 57.27630285 |
| Clupeidae | 15.71 | 435300 | 124800 | 22092.45893 | 31147.33698 | 16984.19612 | 116.9736187 | 80.51269288 |
| Clupeidae | 15.82 | 434700 | 126400 | 21721.38256 | 30655.35183 | 16796.95243 | 75.19821606 | 61.57086224 |
| Clupeidae | 18.4 | 459300 | 113500 | 21328.24143 | 30135.37009 | 16788.99652 | 60.48186786 | 50.54644809 |
| Engraulidae | 9.74 | 437151.9175 | 123593.7442 | 14518.29859 | 22744.78914 | 4561.924382 | 119.1226369 | 93.31071255 |
| Engraulidae | 15.26 | 415607.2645 | 118518.6072 | 9979.405891 | 15725.16226 | 3072.890664 | 88.86669995 | 73.63954069 |
| Engraulidae | 11.34 | 409872.2975 | 120298.7012 | 18285.1291 | 27897.21946 | 8691.658391 | 93.34657398 | 75.84409136 |
| Engraulidae | 11.59 | 438152.9145 | 129985.3222 | 14310.15316 | 22139.32806 | 6100.543478 | 94.12055336 | 69.04644269 |
| Engraulidae | 22.68 | 438100 | 118200 | 16451.25554 | 24976.61251 | 12445.22403 | 32.00393895 | 50.3446578 |
| Engraulidae | 21.97 | 454500 | 123200 | 14212.32877 | 20123.99622 | 14250.11809 | 32.12092584 | 46.41001417 |
| Engraulidae | 99.7 | 454200 | 127600 | 15942.54511 | 21540.83571 | 10193.49478 | 18.39981007 | 40.00474834 |
| Engraulidae | 105.72 | 455700 | 119200 | 13352.83865 | 19392.43028 | 11886.20518 | 24.02888446 | 40.83665339 |
| Engraulidae | 15.12 | 465283.1745 | 89654.81417 | 12199.92636 | 19991.40893 | 4674.766814 | 43.20078547 | 62.10112911 |
| Engraulidae | 23.51 | 508006.8005 | 103186.4622 | 17785.20286 | 23938.54415 | 7801.909308 | 26.37231504 | 43.55608592 |
| Engraulidae | 16.33 | 453514.6105 | 81508.86317 | 11167.1169 | 17773.82122 | 4091.355599 | 49.48428291 | 55.86935167 |
| Nemipteridae | 28.51 | 442726.6028 | 113518.7612 | 20762.04523 | 28687.31563 | 9458.579154 | 50.63913471 | 36.87315634 |
| Nemipteridae | 25.69 | 407887.4518 | 119295.0422 | 23450.17526 | 35822.48373 | 10604.03105 | 47.57135704 | 36.68002003 |
| Nemipteridae | 32.59 | 429841.3178 | 112433.6082 | 19457.92079 | 29585.39604 | 8069.925743 | 62.0049505 | 27.47524752 |
| Nemipteridae | 29.77 | 430055.0178 | 123875.8132 | 28433.75 | 43287.5 | 11413.125 | 42.125 | 29.375 |
| Nemipteridae | 80.34 | 431000 | 105000 | 17352.50372 | 25193.35647 | 8889.439762 | 67.17897868 | 33.46554289 |
| Nemipteridae | 50.58 | 403100 | 97400 | 20794.56415 | 31747.0617 | 7519.588639 | 45.7884427 | 41.625857 |
| Nemipteridae | 27.13 | 411200 | 107300 | 21509.45744 | 34558.23793 | 7593.330015 | 66.94873071 | 38.20308611 |
| Nemipteridae | 43.85 | 472800 | 110100 | 17460.21058 | 28425.56317 | 8347.208619 | 28.89324192 | 24.85308521 |
| Nemipteridae | 40.18 | 422600 | 99500 | 31345.36456 | 56582.56881 | 11417.18976 | 60.96088846 | 39.23225495 |
| Nemipteridae | 42.31 | 445000 | 108200 | 22928.90936 | 39925.2988 | 9609.063745 | 43.57569721 | 29.6314741 |
| Nemipteridae | 40.59 | 424400 | 103900 | 25157.02582 | 43486.84211 | 9977.656405 | 84.78152929 | 30.03972195 |
| Nemipteridae | 50.7 | 434400 | 101500 | 23932.30433 | 42431.93972 | 9355.858046 | 24.79338843 | 31.96402528 |
| Nemipteridae | 53.68 | 453600 | 117300 | 17641.9768 | 28606.81489 | 6606.452392 | 32.8661189 | 24.04543258 |
| Nemipteridae | 40.3 | 436800 | 108100 | 23385.34019 | 42853.6466 | 5135.829662 | 30.34752814 | 25.69750367 |
| Nemipteridae | 41.26 | 438200 | 99600 | 22134.22233 | 46535.39382 | 4869.142572 | 65.42871386 | 20.43868395 |
| Nemipteridae | 267.4 | 448400 | 105600 | 20877.22552 | 35410.48467 | 5008.036597 | 45.25222552 | 22.5024728 |
| Nemipteridae | 42.23 | 444300 | 117100 | 17709.86661 | 28012.87948 | 4887.304508 | 48.06807728 | 22.53909844 |
| Sciaenidae | 108.04 | 466700 | 120700 | 16623.82017 | 25472.5534 | 11711.37606 | 63.58668654 | 35.27074019 |
| Sciaenidae | 90.63 | 481600 | 112900 | 15790.42289 | 25031.71642 | 10615.67164 | 50.99502488 | 31.71641791 |
| Sciaenidae | 70.72 | 478100 | 103300 | 16164.75918 | 29786.5999 | 10670.00477 | 39.34191702 | 31.83118741 |
| Sciaenidae | 44.72 | 448200 | 113900 | 17135.67223 | 31311.33464 | 11322.37488 | 46.00098135 | 30.05397448 |
| Sciaenidae | 78.91 | 490400 | 103300 | 14302.77913 | 22732.81326 | 10245.00244 | 43.88103364 | 27.79132131 |
| Sciaenidae | 63.11 | 434200 | 105500 | 18151.875 | 38917.5 | 10591.875 | 38.125 | 22.75 |
| Sciaenidae | 68.65 | 446500 | 110900 | 19795.07182 | 39839.02922 | 11043.21446 | 59.93065874 | 25.50767707 |
| Sciaenidae | 79.79 | 464100 | 106400 | 15619.38003 | 32168.71746 | 9073.000473 | 28.63227638 | 20.58684335 |
| Sciaenidae | 75.42 | 456600 | 105900 | 15674.10932 | 33940.9468 | 10301.36652 | 36.11517814 | 35.62713519 |
| Sciaenidae | 37.67 | 449100 | 128600 | 13387.5998 | 24754.24152 | 11212.57485 | 47.90419162 | 28.81736527 |
| Sciaenidae | 28.5 | 441300 | 126700 | 14158.63007 | 22864.73788 | 11533.13551 | 110.7814045 | 31.28090999 |
| Sciaenidae | 21.09 | 426000 | 125200 | 17110.02957 | 28755.5446 | 9829.9655 | 39.92114342 | 35.23903401 |
| Sciaenidae | 20.64 | 444900 | 129500 | 15238.18408 | 26402.98507 | 9263.681592 | 38.43283582 | 34.82587065 |
| Sciaenidae | 37.96 | 422400 | 97500 | 22268.58782 | 54304.51597 | 5746.007984 | 50.5239521 | 49.4011976 |
| Sciaenidae | 46.38 | 436300 | 100100 | 20977.40316 | 44315.51889 | 5001.7934 | 70.89909134 | 43.63940698 |
| Sciaenidae | 32.65 | 411300 | 107200 | 21162.13858 | 50374.5015 | 6061.814556 | 87.36291127 | 48.47956132 |
| Sciaenidae | 31.57 | 421900 | 105300 | 22982.50978 | 55339.40802 | 5595.034247 | 57.2407045 | 53.81604697 |
| Sciaenidae | 29.6 | 434700 | 114900 | 21517.37309 | 35939.50222 | 12440.85757 | 49.65500246 | 58.77279448 |
| Sciaenidae | 28.13 | 441500 | 106700 | 23705.88966 | 40486.45626 | 10209.99006 | 51.06858847 | 42.1222664 |
| Sciaenidae | 36.87 | 448500 | 115600 | 18175.42331 | 25894.54681 | 13865.78685 | 46.812749 | 42.33067729 |
| Sciaenidae | 28.28 | 440700 | 115200 | 19080.35271 | 32996.15002 | 13415.30055 | 56.25931446 | 39.61748634 |
| Sciaenidae | 27.44 | 430500 | 110300 | 21122.6624 | 37965.67421 | 11302.90354 | 98.30216535 | 53.1496063 |
| Sciaenidae | 117.11 | 401400 | 113500 | 23194.51161 | 42715.18375 | 11585.46905 | 45.21276596 | 42.67408124 |
| Sciaenidae | 63.76 | 457400 | 99700 | 15434.32725 | 20704.78068 | 10070.84771 | 34.13011336 | 44.72646624 |
| Sciaenidae | 72.21 | 401300 | 109700 | 19934.47329 | 39246.1308 | 11206.31553 | 34.19870195 | 37.69345981 |
| Sciaenidae | 53.88 | 419200 | 112600 | 18850.56762 | 32291.46101 | 11128.45508 | 28.6278381 | 38.74629812 |
| Sciaenidae | 33.1 | 409400 | 113500 | 15921.60494 | 25807.40741 | 8208.024691 | 46.91358025 | 31.11111111 |
| Ophichthidae | 202.54 | 486000 | 115200 | 8201.505429 | 7100.197433 | 10301.70286 | 21.84106614 | 39.11648569 |
| Ophichthidae | 116.68 | 471000 | 100100 | 7667.118895 | 6658.855451 | 7786.753823 | 24.17365565 | 33.05377405 |
| Ophichthidae | 110.83 | 500200 | 115500 | 9698.533068 | 10410.24366 | 8712.705122 | 24.61461959 | 35.05718548 |
| Ophichthidae | 213.52 | 481700 | 108800 | 7705.97639 | 6620.757501 | 8175.725529 | 23.11854402 | 39.84259715 |
| Ophichthidae | 60.46 | 462000 | 127100 | 8918.350168 | 9366.281866 | 7886.604137 | 23.32852333 | 35.11303511 |
| Ophichthidae | 262.34 | 500400 | 109700 | 9922.598732 | 9422.842516 | 10104.82691 | 34.73915163 | 30.59483179 |
| Ophichthidae | 231.43 | 511100 | 112800 | 8943.944553 | 7648.95428 | 9022.373541 | 56.1770428 | 33.68190661 |
| Ophichthidae | 273.47 | 517200 | 104300 | 7712.985437 | 5795.509709 | 8501.213592 | 22.33009709 | 26.09223301 |
| Ophichthidae | 443.36 | 480400 | 118300 | 8341.972035 | 5991.441659 | 10143.44262 | 40.50144648 | 38.33172613 |
| Ophichthidae | 237.43 | 516600 | 113400 | 8826.642336 | 7026.155718 | 9598.540146 | 47.68856448 | 29.31873479 |
| Ophichthidae | 126.08 | 515000 | 118100 | 9801.247554 | 7295.74364 | 14406.18885 | 26.0518591 | 22.87181996 |
| Ophichthidae | 176.15 | 520400 | 109900 | 9703.316953 | 7997.542998 | 12645.5774 | 24.93857494 | 21.4987715 |
| Ophichthidae | 182.46 | 513900 | 122000 | 9707.167832 | 6980.519481 | 14514.86014 | 20.35464535 | 19.23076923 |
| Ophichthidae | 270.27 | 521700 | 115900 | 8784.756098 | 5707.317073 | 13418.90244 | 46.82926829 | 22.80487805 |
| Ophichthidae | 190.36 | 508100 | 115600 | 9644.698085 | 6842.169858 | 14112.05204 | 28.22778596 | 21.23220422 |
| Trichiuridae | 202.54 | 486000 | 115200 | 8201.505429 | 7100.197433 | 10301.70286 | 21.84106614 | 39.11648569 |
| Trichiuridae | 43.39 | 540900 | 87100 | 11440.59406 | 15167.07921 | 4462.252475 | 26.85643564 | 23.14356436 |
| Trichiuridae | 39.21 | 525900 | 104900 | 12104.48497 | 16356.5796 | 4779.448004 | 20.82306555 | 24.51946772 |
| Trichiuridae | 68.93 | 534800 | 86000 | 11280.75253 | 15328.02701 | 5472.744814 | 21.58707188 | 19.05451037 |
| Trichiuridae | 48.5 | 481400 | 104500 | 17632.80866 | 26893.75307 | 5636.98967 | 64.92867683 | 36.0304968 |
| Trichiuridae | 156.44 | 510000 | 105000 | 12500.61607 | 17034.25333 | 8965.007393 | 149.8275012 | 34.37654017 |
| Trichiuridae | 126.85 | 564500 | 75700 | 9551.456071 | 13838.84501 | 7002.714709 | 71.56959526 | 21.84106614 |
| Trichiuridae | 80.43 | 550400 | 85700 | 10914.23767 | 16446.18834 | 7052.130045 | 27.80269058 | 23.20627803 |
| Trichiuridae | 120.63 | 560400 | 87800 | 13685.48387 | 19727.04715 | 7901.985112 | 51.24069479 | 20.09925558 |
| Trichiuridae | 42.02 | 466300 | 113300 | 14609.48995 | 19691.02501 | 4290.093183 | 41.44188328 | 20.47572339 |
| Trichiuridae | 49.5 | 549000 | 93700 | 10513.67461 | 15119.46242 | 3262.817322 | 33.7232454 | 28.99452464 |
| Trichiuridae | 36.22 | 498900 | 119300 | 11891.3097 | 16278.92664 | 3962.333826 | 24.61841457 | 29.29591334 |
| Trichiuridae | 22.36 | 482800 | 119000 | 13718.92093 | 20394.08255 | 1833.664843 | 66.01193436 | 36.05171556 |
| Carangidae | 9.77 | 466900 | 120600 | 14340.55611 | 15482.21443 | 9817.760521 | 52.47995992 | 30.81162325 |
| Carangidae | 9.1 | 441300 | 116700 | 15738.07768 | 17079.64602 | 10334.93117 | 43.633235 | 33.18584071 |
| Carangidae | 11.08 | 463500 | 119300 | 14577.56061 | 15241.21722 | 8508.782781 | 51.95447798 | 33.52300841 |
| Carangidae | 8.69 | 440800 | 116300 | 13549.375 | 14188.75 | 9116.875 | 55.125 | 40.625 |
| Carangidae | 7.43 | 451400 | 116000 | 13409.03465 | 14549.50495 | 7801.361386 | 68.44059406 | 33.41584158 |
| Carangidae | 75.84 | 416400 | 107500 | 16733.77257 | 22335.28551 | 9324.060517 | 60.39531479 | 46.60810151 |
| Carangidae | 98.92 | 444400 | 107300 | 16334.88031 | 21636.54128 | 10304.10357 | 62.16414265 | 49.21836834 |
| Carangidae | 120.82 | 430900 | 108800 | 17560.10795 | 25289.49951 | 9552.257115 | 62.80667321 | 40.48086359 |
| Carangidae | 148.77 | 417600 | 108100 | 16068.78698 | 22048.81657 | 9063.116371 | 76.79980276 | 43.76232742 |
| Carangidae | 196.54 | 402400 | 108000 | 13067.62695 | 17121.58203 | 8388.671875 | 85.32714844 | 46.75292969 |
| Carangidae | 42.32 | 466100 | 111100 | 13479.09451 | 16432.45918 | 8780.925285 | 54.3047996 | 45.64571994 |
| Carangidae | 45.7 | 467900 | 107000 | 12619.31256 | 14359.545 | 8523.120673 | 42.28486647 | 47.23046489 |
| Carangidae | 27.76 | 473100 | 104200 | 18108.87897 | 27614.0873 | 15646.08135 | 52.33134921 | 48.61111111 |
| Carangidae | 152.01 | 482000 | 104100 | 15471.74447 | 20159.70516 | 10178.13268 | 43.12039312 | 43.73464373 |
| Carangidae | 21.7 | 457500 | 113900 | 17018.53635 | 23326.91382 | 10748.67597 | 52.47953779 | 59.70149254 |
| Carangidae | 312.71 | 460300 | 114400 | 18607.80263 | 23420.63685 | 12870.68546 | 84.46767137 | 59.55274672 |
| Carangidae | 211.93 | 459680.2404 | 141928.925 | 14856.64854 | 16034.35492 | 10090.83045 | 101.3346515 | 79.09045971 |
| Carangidae | 147.49 | 481127.1194 | 124759.821 | 12682.111 | 13553.02279 | 6337.338949 | 121.407334 | 30.84737364 |
| Carangidae | 201.84 | 477403.5404 | 129055.532 | 12026.50602 | 11644.57831 | 8458.433735 | 96.38554217 | 29.15662651 |
| Carangidae | 159.94 | 486505.9464 | 125086.145 | 12247.63799 | 14116.11139 | 6576.951765 | 77.69766285 | 30.08453506 |
| Carangidae | 144.69 | 471151.4254 | 127714.87 | 10714.98771 | 9993.857494 | 6855.651106 | 94.59459459 | 36.97788698 |
| Carangidae | 43.03 | 434870.7424 | 116505.422 | 13822.44597 | 17669.4499 | 6674.238703 | 165.1522593 | 47.2740668 |
| Carangidae | 49.09 | 449688.1024 | 113698.531 | 10614.24474 | 13602.39163 | 6169.033383 | 366.8410563 | 34.1305431 |
| Carangidae | 45.92 | 471680.4934 | 121085.948 | 17306.35551 | 12446.22371 | 7451.601338 | 147.5860421 | 47.20363289 |
| Carangidae | 47.27 | 458290.7204 | 123112.426 | 15058.45771 | 26290.96326 | 8671.176763 | 76.34061569 | 46.30089374 |
| Carangidae | 171.71 | 489600 | 138000 | 15457.40741 | 20982.58706 | 8688.432836 | 152.3631841 | 51.99004975 |
| Carangidae | 172.95 | 453800 | 123200 | 17384.96555 | 25123.64665 | 7904.773622 | 65.82185039 | 37.89370079 |
| Carangidae | 103.19 | 491300 | 119400 | 13423.61458 | 17081.87718 | 6729.905142 | 57.16425362 | 48.67698452 |
| Carangidae | 157.28 | 432340.2176 | 119998.0008 | 15520.33205 | 31620.05374 | 6240.840254 | 46.89789936 | 43.84465071 |
| Carangidae | 165.74 | 469900 | 116200 | 17175.43436 | 25097.73166 | 8363.899614 | 62.74131274 | 42.3503861 |
| Carangidae | 92.72 | 492200 | 111600 | 15321.79425 | 20058.50804 | 7248.902974 | 50.09751341 | 26.08483667 |
| Carangidae | 56.69 | 426168.0864 | 126275.933 | 16795.12195 | 24219.5122 | 6500.609756 | 61.58536585 | 62.19512195 |
| Carangidae | 35.25 | 457558.1914 | 127426.489 | 15625.94079 | 20459.10687 | 6504.641244 | 62.71951831 | 47.79227296 |
| Carangidae | 43.84 | 478158.4704 | 122706.379 | 13401.5984 | 16314.93506 | 5829.795205 | 69.30569431 | 37.33766234 |
| Carangidae | 40.84 | 453965.3024 | 116208.514 | 15490.53785 | 20312.5 | 5554.656375 | 46.68824701 | 46.937251 |
| Carangidae | 46.9 | 473649.6584 | 120325.105 | 12013.37296 | 11589.89599 | 5639.549282 | 48.91035166 | 37.51857355 |
| Carangidae | 57.52 | 470335.2734 | 119158.827 | 16202.29941 | 21777.15264 | 7326.932485 | 65.80234834 | 41.34050881 |
| Carangidae | 69.19 | 445458.7184 | 118357.248 | 14906.7345 | 19876.45349 | 6910.731589 | 123.6676357 | 32.46124031 |
| Carangidae | 48.09 | 450005.6334 | 135414.809 | 17518.69392 | 23398.55434 | 9347.582253 | 58.32502493 | 36.2662014 |
| Gobiidae | 17.25 | 414500 | 122600 | 18212.03299 | 27899.07812 | 12536.3901 | 85.63803979 | 54.94905386 |
| Gobiidae | 18.68 | 417000 | 123000 | 20186.92716 | 34424.74674 | 12180.41486 | 28.70236372 | 50.16883743 |
| Gobiidae | 12.47 | 409400 | 122500 | 19382.38189 | 33113.92717 | 11577.26378 | 111.2204724 | 49.70472441 |
| Gobiidae | 13.5 | 423400 | 125300 | 20121.00798 | 33751.2475 | 12275.4491 | 32.80938124 | 44.41117764 |
| Gobiidae | 21.14 | 436800 | 127300 | 15471.52367 | 24081.6075 | 7102.440828 | 134.9852071 | 33.28402367 |
| Gobiidae | 23.98 | 437800 | 127600 | 12874.87525 | 17309.13174 | 6947.979042 | 88.32335329 | 30.93812375 |
| Gobiidae | 18.27 | 392900 | 118100 | 26634.0399 | 48927.6808 | 6670.822943 | 96.3840399 | 33.29177057 |
| Gobiidae | 23.33 | 382500 | 107900 | 21564.98509 | 38301.44135 | 4855.864811 | 105.9890656 | 37.027833 |
| Gobiidae | 10.43 | 422000 | 122100 | 19265.76465 | 32305.1142 | 6081.181728 | 106.0079444 | 32.14995035 |
| Gobiidae | 10.98 | 422500 | 131500 | 14171.69402 | 24644.06371 | 4473.938224 | 50.07239382 | 36.1969112 |
| Gobiidae | 14.18 | 433800 | 133600 | 8775.647289 | 14099.9023 | 4992.672203 | 53.73717636 | 27.96775769 |
| Gobiidae | 7.54 | 432600 | 132900 | 10184.42623 | 20727.7695 | 4385.245902 | 49.42871336 | 27.69498261 |
| Gobiidae | 13.14 | 425500 | 131000 | 10863.19822 | 18209.34256 | 4439.569946 | 33.98418191 | 33.36628769 |
| Gobiidae | 12.59 | 429000 | 132200 | 10848.85743 | 17343.51714 | 4395.802285 | 34.15300546 | 28.31594635 |
| Gobiidae | 17.82 | 454600 | 133500 | 9241.157946 | 10660.12597 | 12498.18314 | 40.69767442 | 30.28100775 |
| Gobiidae | 25.51 | 451300 | 131800 | 9812.70943 | 10496.64911 | 12587.96075 | 59.23887027 | 31.35471517 |
| Gobiidae | 18.25 | 446700 | 133100 | 11000.12494 | 14356.57171 | 11323.71314 | 71.71414293 | 28.36081959 |
| Gobiidae | 9.61 | 442100 | 125700 | 17065.18357 | 33238.95457 | 11455.35159 | 88.51897946 | 41.38145613 |
| Gobiidae | 9 | 421200 | 126700 | 12320.54761 | 20037.00049 | 10191.78589 | 40.82387765 | 35.64380858 |
| Gobiidae | 16.05 | 421700 | 127400 | 14386.71105 | 28205.12821 | 7062.376726 | 32.05128205 | 46.59763314 |
| Gobiidae | 19.26 | 431600 | 126000 | 17280.40541 | 24130.38038 | 6587.837838 | 78.07807808 | 34.28428428 |
| Gobiidae | 12.08 | 429600 | 129600 | 12699.5276 | 22799.60219 | 5390.353058 | 19.51765291 | 41.64594729 |
| Gobiidae | 13.28 | 418500 | 121500 | 13264.78628 | 24496.76938 | 7164.512922 | 44.85586481 | 50.32306163 |
| Gobiidae | 6.47 | 444000 | 130400 | 10890.70681 | 19810.20942 | 5266.361257 | 32.19895288 | 32.85340314 |
| Gobiidae | 20.62 | 451800 | 133100 | 12668.46192 | 16346.44289 | 9899.173347 | 26.42785571 | 27.30460922 |
| Gobiidae | 16.56 | 457800 | 133400 | 10647.29931 | 12736.62042 | 10200.07433 | 32.08622398 | 27.5024777 |
| Gobiidae | 19.56 | 432300 | 126400 | 16413.33826 | 24859.46746 | 9650.517751 | 52.5147929 | 29.09270217 |
| Gobiidae | 15.67 | 457400 | 133100 | 11708.25219 | 15245.86173 | 9564.873418 | 41.50438169 | 29.33300876 |
| Gobiidae | 12.85 | 458100 | 134400 | 10791.999 | 13678.96311 | 9151.919242 | 38.50947159 | 29.16251246 |

**Multidimensional stoichiometric niche code：**

rm(list=ls())

install.packages("hypervolume")

# install other packages that you need for normal analyses, such as PCA...

#input elemental contents of earthworms, millipedes, and litters

getwd()

setwd("D:/R2")

data<-read.csv("STW.csv")

pctx.ind.all <- read.csv("STW.csv", header = TRUE)

head(pctx.ind.all)

taxon.list <- c("Leiognathidae","Siganidae","Clupeidae","Engraulidae","Ophichthidae","Nemipteridae","Trichiuridae","Sciaenidae","Carangidae","Gobiidae")

pctx.ind.all$taxon <- factor(pctx.ind.all$taxon,levels=taxon.list)

table(pctx.ind.all$site, pctx.ind.all$taxon)

pctx.ind.dt.lt <- pctx.ind.all[!is.na(pctx.ind.all$PctC),-3]

table(pctx.ind.dt.lt$site, pctx.ind.dt.lt$taxon)

asq.ind.dt.lt <- data.frame(pctx.ind.dt.lt[,c(1:2)], asin(sqrt(pctx.ind.dt.lt[,c(3:9)]/100)))#74litter,76 detritivores

colnames(asq.ind.dt.lt)[-c(1:2)] <- c("C","N","P","Ca","K","Fe","Zn")

summary(asq.ind.dt.lt)

asq.ind.dt.lt$taxon <- factor(asq.ind.dt.lt$taxon, levels=taxon.list)

pca_asq.ind.dt.lt <- stats::prcomp(asq.ind.dt.lt[,c(3:9)], scale=TRUE)

(pca_asq.ind.dt.lt_result <- summary(pca_asq.ind.dt.lt))

pca123 <- data.frame(asq.ind.dt.lt[,c(1:2)], pca_asq.ind.dt.lt_result$x[,c(1:3)])

head(pca123)

(var.infor.ind.dt.lt <- data.frame(pca_asq.ind.dt.lt_result$rotation[,1:3]))

library(plyr)

group_border.ind.dt.lt <- ddply(pca123, c('taxon'),function(df) df[chull(df[["PC1"]],df[["PC2"]]), ])

pca123$taxon <- factor(pca123$taxon, levels=taxon.list)

library(ggplot2)

library(ggrepel)

library(tidyr)

pca.asq.ind.dt.lt.fig <-

ggplot()+

geom_point(data=pca123, aes(x=PC1,y=PC2, color = taxon, shape=taxon),size=3)+

scale_shape_manual(values = c(18,19,25,17,7,13,23,12,15,9))+

scale_color_manual(values = c("#A020F0","#FF7F00","#7caf2a","#CD1076","#ffe327","#2f4e87","#db6968","#4593c3","#187c65","#a2a7ab")) +

geom_segment(data=var.infor.ind.dt.lt,aes(x=0,y=0,xend=PC1*10,yend=PC2*10),

arrow = arrow(angle = 22.5,length = unit(0.25,"cm"), type="closed"), linetype=1, size=0.5, color="black")+

geom_text_repel(data=var.infor.ind.dt.lt,aes(x=PC1*10,y=PC2*10), label=row.names(var.infor.ind.dt.lt), size =6)+

geom_polygon(data = group_border.ind.dt.lt, aes(PC1, PC2, color = taxon, fill=taxon), alpha = 0.3)+

scale_fill_manual(values = c("#A020F0","#FF7F00","#7caf2a","#CD1076","#ffe327","#2f4e87","#db6968","#4593c3","#187c65","#a2a7ab")) +

geom_vline(xintercept = 0, color = 'black', linetype="dashed",size = 1) +

geom_hline(yintercept = 0, color = 'black', linetype="dashed",size = 1) +

labs(x=paste("PC1 (", format(100 *pca_asq.ind.dt.lt_result$importance[2,1], digits=3), "%)", sep=""),

y=paste("PC2 (", format(100 *pca_asq.ind.dt.lt_result$importance[2,2], digits=3), "%)", sep=""))+

theme(panel.grid = element_line(color = 'gray', linetype = 2, size = 0.1),

panel.background = element_rect(color = 'black', fill = 'transparent'),

legend.key = element_rect(fill = 'transparent'),

axis.title.x = element_text(size = 24, color = "black", vjust = 0.5, hjust = 0.5),

axis.title.y = element_text(size = 24, color = "black", vjust = 0.5, hjust = 0.5),

axis.text.x = element_text(size = 20, color = "black", vjust = 0.5, hjust = 0.5, angle = 0),

axis.text.y = element_text(size = 20, color = "black", vjust = 0.5, hjust = 0.5),

legend.position = "top", legend.box="vertical",

legend.title = element_text(colour="black", size=24),

legend.text = element_text(colour="black", size=12),

legend.background = element_rect(fill="transparent",size= 0.4, linetype="solid"))

pca.asq.ind.dt.lt.fig

####### ~~~~~~~~ Now we calculate hypervolumes for three taxa in three forests ~~~~~~~~ #######

# re-order forest and taxa , hypervolumes will be calculated follow the order of the group/level "id.col" see below.

# combine "forest" and "taxon" to a new column "id.col", resulting in nine groups (3 forests X 3 taxa)

pca123.taxon <- unite(pca123,"id.col",c("taxon"), sep="_", remove = F)

# calculate hypervolume for each group/level in "id.col" using the function "generateHypervolume"

library(hypervolume)

generateHypervolume <- function(data, id.col){

idlist <- as.vector(unique(data$id.col))

est_bw <- estimate_bandwidth(data[,! names(data) %in% id.col])

res <- list()

for(i in 1:length(idlist)){

res[[idlist[i]]] <- hypervolume(data = dplyr::select(dplyr::filter(data, id.col %in% idlist[i]),-id.col))

}

return(res)

}

# calculating hypervolume for each group/level in the "id.col", put all results in a list

hpv.taxon <- generateHypervolume(pca123.taxon[, c("id.col","PC1","PC2","PC3")],"id.col")

# extract hypervolume size, calculate overlap and distance for each pair of group/level in "id.col"

pairwiseHypervolumeOverlap <- function(list, method){ #list = list of hypervolume objects; method = "sorensen", "jaccard"

hypervol_dim <- length(list)

res <- res.dist <- matrix(NA, nrow = hypervol_dim, ncol = hypervol_dim)

colnames(res) <- colnames(res.dist) <- names(list)

rownames(res) <- rownames(res.dist) <- names(list)

pairs <- t(combn(1:hypervol_dim, 2))

overlap <- vector()

hv.dist <- vector()

for(i in 1:nrow(pairs)){

hv1 <- list[[pairs[i,][1]]]

hv2 <- list[[pairs[i,][2]]]

set <- hypervolume_set(hv1, hv2, check.memory = FALSE, num.points.max = 10^6)

overlap[i] <- hypervolume_overlap_statistics(set)[method]

hv.dist[i] <- hypervolume_distance(hv1, hv2, type = "centroid", num.points.max = 1000, check.memory = TRUE)

}

res[lower.tri(res)] <- overlap

res.dist[lower.tri(res.dist)] <- hv.dist

res.dist.t <- t(res.dist)

res[upper.tri(res)] <- res.dist.t[upper.tri(res.dist.t)]

res <- data.frame(res)

res$hypervolume <- as.vector(sapply(list, get_volume))

return(res)

}

hpv_overlap.taxon <- pairwiseHypervolumeOverlap(hpv.taxon,"jaccard")
